# Trends in chlamydia prevalence in the United States, 2005–2016

**DOI:** 10.1038/s41598-024-61818-5

**Published:** 2024-05-23

**Authors:** Yu Cheng, Guanghao Zheng, Zhen Song, Gan Zhang, Xuepeng Rao, Tao Zeng

**Affiliations:** The Second Affiliated Hospital, Jiangxi Medical College, Nanchang University, No. 1, Minde Road, Nanchang, 330006 Jiangxi China

**Keywords:** Chlamydia prevalence, Trends, NHANES, Risk factors, Risk factors, Urology

## Abstract

In the United States (US), chlamydia is the most frequently reported sexually transmitted infection that is nationally notifiable. We examined trends in chlamydia prevalence in the US in 2011–2016 compared with 2005–2010. Cross-sectional, nationally representative surveys, National Health and Nutrition Examination Surveys (NHANES), were used to compare national chlamydia prevalence estimates from 2011 to 2016 with those from 2005 to 2010, and changes in prevalence since 1999–2004 were also reviewed. Persons aged 18–39 years were included in these analyses. Prevalence of chlamydia was based on results from urine specimens. Results were weighted to represent the U.S. civilian, noninstitutionalized population. The baseline characteristics of the study population were similar in gender, age and race/ethnicity between the two groups (*P* > 0.05). The overall chlamydia prevalence was 1.88% (95% confidence interval [CI] 1.55–2.22%) in 2011–2016 and 1.57% (95% CI 1.27–1.87%) in 2005–2010, a relative increase of 19.7% (95% CI 0.2–39.2%; *P* < 0.05) between the two surveys. Increases in chlamydia prevalence was especially concentrated in persons who were male, aged 18 to 29 years, had > high school educational level, never married, age at first sex < 18 years, had 2–5 sexual partners in lifetime and had no past sexually transmitted diagnosis between 2005 and 2016 (*P* < 0.05). Multivariable logistic regression analysis demonstrated that chlamydia was more prevalent in those aged 18–29 years, being non-Hispanic Blacks, had high school educational level, being widowed/divorced/separated and had > 5 sexual partners. The chlamydia prevalence had an increasing trend from 2005–2010 to 2011–2016. Those with high chlamydia prevalence such as sexually active young adults and Non-Hispanic Black should be screened annually so that infected persons can be diagnosed and they and their sex partners can be treated promptly.

## Introduction

Caused by the infection with *Chlamydia trachomatis*, chlamydia is the leading notifiable disease in the United States (US). It ranks among the most widespread sexually transmitted infection (STI), with the highest proportion of reported STI cases to CDC since 1994^1^. Chlamydial infections commonly show no symptoms in men and women^2^. However, if left untreated, these infections can lead to acute urogenital diseases like urethritis, vaginitis, cervicitis, and genital ulceration in men or women^3,4^. Similar to other inflammatory STI, chlamydial infection can increase the risk of HIV transmission^5^. Moreover, newborns with congenital chlamydial infection via in utero transmission had increased risk of ophthalmia neonatorum, a condition that can result in blindness, as well as pneumonia^6^.

Chlamydia infection imposes a substantial cost burden on medical care in the United States. In the study conducted by Owusu-Edusei Jr. and colleagues, the total direct medical costs for chlamydia in 2008 were estimated to be $516.7 million for individuals aged 15–39 years^7^. The study further revealed that among nonviral STI, chlamydia was the most expensive infection. It emphasized that the burden would be even higher in the absence of efforts for STI prevention and control.

There have been two studies that showed the high prevalence of chlamydial infections in the general, non-institutionalized population of the United States, with a particular emphasis on young women^8,9^. Datta et al. reported that the prevalence of Chlamydia infection was 2.2% in 1999–2002 among persons aged 14–39 years about two decades ago and Torrone et al. reported a relative decreased prevalence of Chlamydia infection in 2007–2012, with an estimate of 1.7% about a decade ago. However, the recent prevalence and trends of chlamydia infection in US remain unclear. We used nationally representative data from the National Health and Nutrition Examination Survey (NHANES) to evaluate the prevalence and trends of chlamydia infection in 2005–2010 and 2011–2016 among US adults and to further determine the correlates of chlamydia infection.

## Methods

### Study population and survey design

The NHANES is a program of surveys conducted by the National Center for Health Statistics (NCHS) to collect health-related data from the civilian noninstitutionalized population in the US. These surveys use a complex and multistage probability sample design to establish a representative sample of the population. The aim is to obtain comprehensive information on health and nutrition status through interviews and examinations. The detailed information of study design, protocol, and data collection for NHANES are described in existing publications^10^. From 1999 to March 2020, there were a total of ten cycles for NHANES surveys, consisting of nine 2-year cycles spanning from 1999 to 2016 and one combined cycle from 2017 to March 2020, which was impacted by the COVID-2019 pandemic^11^. Chlamydia prevalence was surveyed from 1999 to 2016 including nine cycles. The present study included adult population aged ≥ 18 years and who had complete data for chlamydia laboratory test and focused on the chlamydia prevalence of last three cycles 2011–2016 and previous three cycles 2005–2010. The unweighted total response rates ranged from 61.3 to 80.5% for the interviewed samples and from 58.7 to 77.4% for the examined samples^12^. All participants included in this study were interviewed and medically examined with CT lab results. For this study, the NHANES protocol obtained approval from the National Center for Health Statistics Ethics Review Board, and all participants provided informed consent.

### Covariates of chlamydia infection

To examine covariates possibly associated with chlamydia infection, we extracted data on gender (male and female), age (18–29 years and 30–39 years), race/ethnicity (Mexican American, other Hispanic, non-Hispanic White, non-Hispanic Black and other), education (< high school, high school and >  high school), marital status (never married, widowed/divorced/separated and married/living with partner), family poverty ratio (<100% and ≥ 100%), age at first sex (< 18 years and ≥ 18 years, sex was defined as vaginal, oral, or anal), number of sexual partners in lifetime (0, 1, 2–5, > 5) and past STI diagnosis (participants who have been told by a doctor or other health care professional in the last 12 months that they had chlamydia or gonorrhea or genital herpes or genital warts).

### Statistical analysis

Statistical analysis was performed with *R* version 4.3.1. Descriptive statistics were generated to show the demographic characteristics and other participants’ features, and the difference of NHANES 2005–2010 and 2011–2016 was compared using Wilcoxon rank-sum test. Estimates on weighted prevalence (95% confidence interval (CI)) of chlamydia were calculated in the two surveys. The percent change (95% CI) was calculated and the differences in prevalence between the surveys were considered to be statistically significant if χ^2^ test had a *P* value of less than 0.05. Additionally, estimates on weighted prevalence (95% CI) of chlamydia were further calculated by gender, age, race/ethnicity, education, marital status, family poverty ratio, age at first sex, lifetime no. sexual partners and past STI diagnosis group, the percent change (95% CI) was also calculated and the difference was also compared between the two surveys in each subgroup. Furthermore, we estimated and compared overall, gender-specific, age-specific and race/ethnicity-specific prevalence of chlamydia in 1999–2004, 2005–2010 and 2011–2016. Last, weighted logistic regressions were used to explore the risk factors of chlamydia infection by incorporating gender, age, race/ethnicity, education, marital status, family poverty ratio, age at first sex, lifetime no. sexual partners and past STI diagnosis by combining NHANES 2005–2010 and 2011–2016. All statistical tests were 2 sided, with *P* < 0.05 considered statistically significant.

### Ethic statement

For this study, the NHANES protocol obtained approval from the National Center for Health Statistics Ethics Review Board, and all participants provided informed consent. All methods were carried out in accordance with relevant guidelines and regulations.

## Results

### Participant characteristics

Of the 60,936 individuals who participated in NHANES from 2005 to 2016, we excluded 24,649 participants who were younger than 18 years and 23,258 who had no chlamydia screening information. The final study population included 13,029 adults with complete data, 6651 in 2005–2010 and 6378 in 2011–2016. There was no significant difference of gender, age and race/ethnicity composition between the two surveys (*P* > 0.05). Detailed data on demographic characteristics and other participants’ features were shown in Table 1.

**Table 1:** Baseline Characteristics of the Study Population in the NHANES 2005–2010 and NHANES 2011-2016^a^.

Characteristic	2005–2010	2011–2016	*P* value^e^
Number	6651	6378	
Weighted (*N*)	255,577,321	266,344,573	
Gender			> 0.05
Male	49.9	50.0	
Female	50.1	50.0	
Age, y			> 0.05
18–29	54.7	55.5	
30–39	45.3	44.5	
Race/ethnicity			> 0.05
Mexican American	12.4	12.7	
Other Hispanic	6.2	8.1	
Non-Hispanic White	61.3	56.3	
Non-Hispanic Black	13.2	12.9	
Other^b^	6.9	10.1	
Education			< 0.05
< High school	17.9	13.8	
High school	22.7	19.8	
> High school	59.4	66.4	
Marital status			
Never married	35.8	37.8	
Widowed/divorced/separated	6.9	6.3	
Married/living with partner	57.3	55.9	
Family poverty ratio			< 0.05
< 100%	18.9	22.4	
≥ 100%	81.1	77.6	
Age at first sex^c^, y			
< 18	64.9	63.0	
≥ 18	35.1	37.0	
Lifetime no. sexual partners			< 0.05
0	3.3	8.2	
1	13.6	15.6	
2–5	32.4	31.6	
> 5	50.8	44.7	
Past STI diagnosis^d^			> 0.05
Yes	9.2	7.8	
No	90.8	92.2	

Data were expressed as %. NHANES National Health and Nutrition Examination Survey, STI sexually transmitted infection.

^a^All estimates were weighted to be nationally representative. No. of participants for some variables may not sum up to equal the unweighted and weighted number due to missing data.

^b^“Other” includes race and ethnicity other than non-Hispanic White, non-Hispanic Black, and Hispanic, including multiracial.

^c^Defined as vaginal, oral, or anal.

^d^Participants who have been told by a doctor or other health care professional in the last 12 months that they had chlamydia or gonorrhea or have ever been told they have herpes or genital warts.

^e^Wilcoxon rank-sum test for complex survey samples.

### Prevalence and trends of chlamydia in 2005–2010 and 2011–2016

The overall chlamydia prevalence was 1.88% (95% CI 1.55%–2.22%) in 2011–2016 and 1.57% (95% CI 1.27%–1.87%) in 2005–2010, a relative increase of 19.7% (95% CI 0.2%–39.2%; *P* < 0.05) between the two surveys (Table 2). Additionally, an increasing trend was observed for male from 1.45% to 1.94% (percent change, 33.8% [95% CI (2.1%–65.4%)]; *P* < 0.05), for younger adults aged 18–29 years from 2.18% to 2.81% (percent change, 28.4% [95% CI (4.5%–52.2%)]; *P* < 0.05), for those who had > high school educational level from 0.94 to 1.34% (percent change, 42.2% [95% CI (2.9%–81.5%)]; *P* < 0.05), for those who were never married from 1.93% to 2.76% (percent change, 43.5% [95% CI (11.2%–75.7%)]; *P* < 0.05), for those aged < 18 years when had the first sex from 1.77% to 2.48% (percent change, 39.6% [95% CI (10.5%–68.7%)]; *P* < 0.05), for those who had 2–5 sexual partners in lifetime from 1.43% to 2.29% (percent change, 60.6% [95% CI (17.1%–104.0%)]; *P* < 0.05) and for those who had no past STI diagnosis from 1.40% to 2.07% (percent change, 47.9% [95% CI (20.6%–75.1%)]; *P* < 0.05).

**Table 2:** Changes in Weighted *Chlamydia trachomatis* Prevalence in Persons Aged 18–39 Years Between NHANES in 2005–2010 and 2011–2016^a^.

	2005–2010	2011–2016	Change, % (95% CI)	*P* value
	Prevalence (95% CI)	Prevalence (95% CI)		
Overall	1.57 (1.27–1.87)	1.88 (1.55–2.22)	19.7 (0.2–39.2)	0.016
Gender				
Male	1.45 (1.03–1.86)	1.94 (1.45–2.42)	33.8 (2.1–65.4)	0.023
Female	1.70 (1.27–2.13)	1.83 (1.37–2.29)	7.7 (-22.3–37.7)	0.070
Age, y				
18–29	2.18 (1.73–2.64)	2.81 (2.27–3.34)	28.4 (4.5–52.2)	0.038
30–39	0.83 (0.49–1.17)	0.73 (0.41–1.05)	− 12.3 (− 65.5–40.6)	0.091
Race/ethnicity				
Mexican American	1.97 (1.28–2.65)	2.67 (1.68–3.67)	35.7 (− 13.2–84.7)	0.173
Other Hispanic	1.95 (0.84–3.05)	2.42 (1.23–3.60)	24.2 (− 48.6–97.1)	0.254
Non-Hispanic White	0.75 (0.42–1.08)	0.95 (0.53–1.36)	26.5 (− 31.3–84.4)	0.154
Non-Hispanic Black	5.03 (3.91–6.14)	5.12 (3.97–6.27)	1.9 (− 29.0–32.8)	0.089
Other^b^	1.26 (0.10–2.41)	1.53 (0.83–2.23)	21.8 (− 67.1–110.6)	0.574
Education				
< High school	1.76 (1.07–2.46)	2.31 (1.35–3.28)	31.1 (− 24.1–86.2)	0.123
High school	2.01 (1.26–2.76)	2.47 (1.58–3.37)	23.1 (− 25.0–71.3)	0.259
> High school	0.94 (0.59–1.30)	1.34 (0.95–1.72)	42.2 (2.9–81.5)	0.048
Marital status				
Never married	1.93 (1.37–2.48)	2.76 (2.07–3.46)	43.5 (11.2–75.7)	0.031
Widowed/divorced/separated	2.81 (1.24–4.37)	3.95 (1.98–5.93)	40.9 (− 32.0–113.7)	0.616
Married/living with partner	0.90 (0.58–1.22)	0.72 (0.41–1.02)	− 20.1 (− 67.3–27.0)	0.334
Family poverty ratio				
< 100%	2.31 (1.59–3.02)	2.61 (1.86–3.36)	13.1 (− 24.5–50.7)	0.215
≥ 100%	1.42 (1.07–1.76)	1.58 (1.20–1.95)	11.3 (− 17.3–39.8)	0.278
Age at first sex^c^, y				
< 18	1.77 (1.31–2.24)	2.48 (1.93–3.02)	39.6 (10.5–68.7)	0.025
≥ 18	0.85 (0.41–1.30)	1.06 (0.56–1.55)	24.1 (− 32.9–81.0)	0.349
Lifetime no. sexual partners				
0	0.33 (− 0.48–1.14)	0.84 (0.08–1.60)	156.2 (− 58.5–370.9)	0.918
1	0.58 (0.02–1.15)	0.42 (0.00–0.84)	− 28.3 (− 121.3–64.7)	0.871
2–5	1.43 (0.85–2.00)	2.29 (1.60–2.99)	60.6 (17.1–104.0)	0.041
> 5	1.70 (1.18–2.22)	2.22 (1.62–2.81)	30.5 (− 4.7–65.8)	0.267
Past STI diagnosis^d^				
Yes	1.96 (0.14–3.29)	0.56 (− 0.14–1.27)	− 71.2 (− 182.4–39.9)	0.772
No	1.40 (1.05–1.75)	2.07 (1.65–2.13)	47. 9 (20.6–75.1)	0.033

Data were expressed as % (95% CI). NHANES National Health and Nutrition Examination Survey, CI confidence interval, STI sexually transmitted infection.

^a^All estimates were weighted to be nationally representative.

^b^“Other” includes race and ethnicity other than non-Hispanic White, non-Hispanic Black, and Hispanic, including multiracial.

^c^Defined as vaginal, oral, or anal.

^d^Participants who have been told by a doctor or other health care professional in the last 12 months that they had chlamydia or gonorrhea or have ever been told they have herpes or genital warts.

### Chlamydia prevalence during 1999–2016

**Figure 1:**
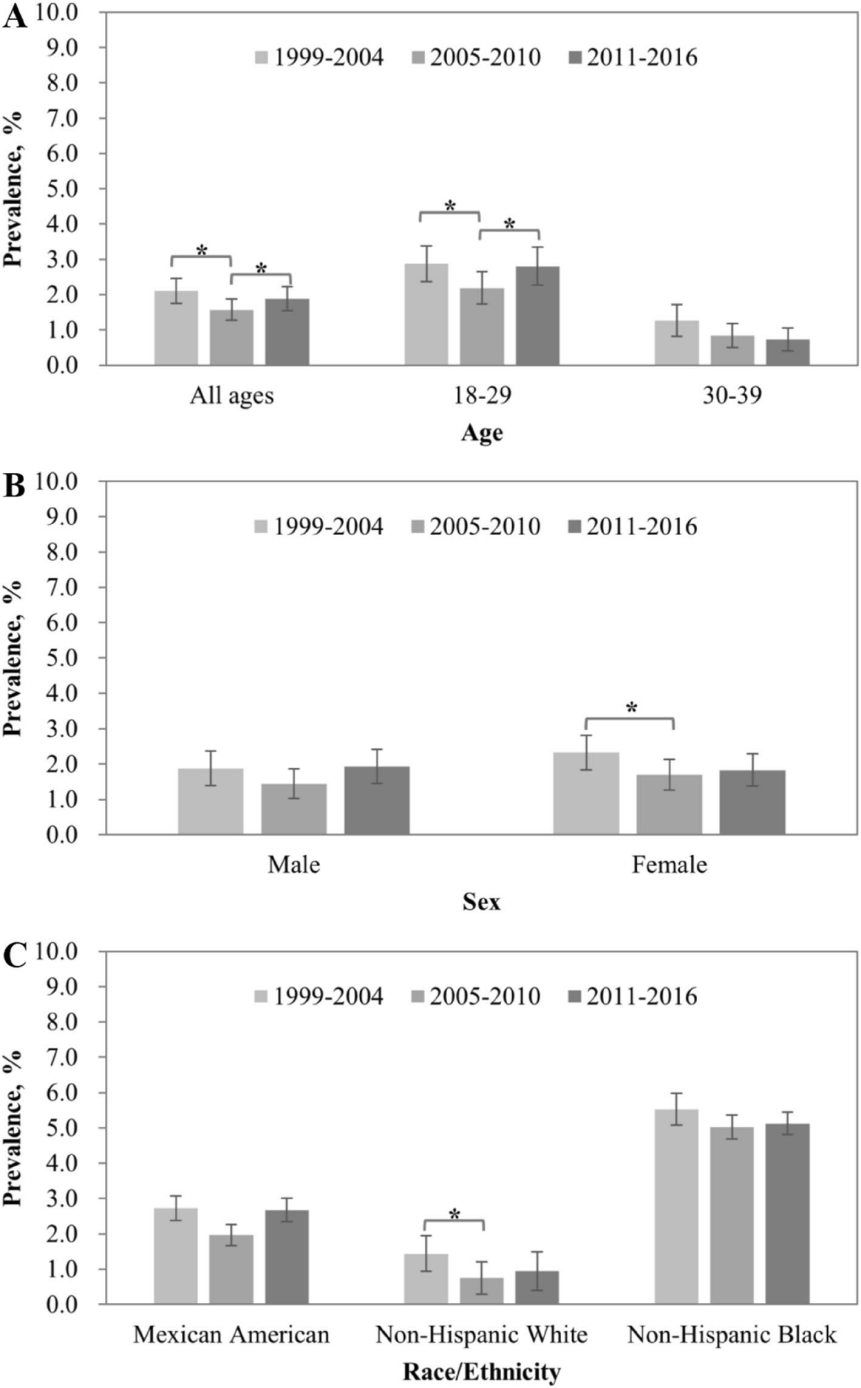
*Chlamydia trachomatis* prevalence by age, sex, race/ethnicity and time period. *P < 0.05

Overall, chlamydia prevalence decreased from 2.10% in 1999–2004 to 1.57% in 2005–2010 (*P* < 0.05) but re-increased to 1.88% in 2011–2016 (*P* < 0.05, Fig. 1A). A similar pattern was observed in younger adults, decreased from 2.87% in 1999–2004 to 2.18% in 2005–2010 (*P* < 0.05) but increased to 2.81% in 2011–2016 (*P* < 0.05). Although chlamydia prevalence had a continuous decrease from 1994–2004 to 2011–2016 in persons aged 30–39 years, the differences were not statistically significant. Additionally, chlamydia prevalence had a significant decrease from 2.32% in 1999–2004 to 1.70% (*P* < 0.05) in 2005–2010 and remained stable in 2011–2016 for female adults (Fig. 1B) and decreased from 1.44% in 1999–2004 to 0.75% (*P* < 0.05) in 2005–2010 and remained stable in 2011–2016 for non-Hispanic White (Fig. 1C). Chlamydia prevalence was stable for male, Mexican American and non-Hispanic Black across the three surveys.

### Risk factors of chlamydia infection

In weighted multivariate logistic regression analysis, when compared to the counterparts in each of subgroup, the prevalence of chlamydia was significantly higher for younger adults (OR 3.28; 95% CI 2.01–5.35) and those who were Mexican American (OR, 2.85; 95% CI 1.72–4.72) or non-Hispanic Black (OR 5.01; 95% CI 3.14–8.01), had an educational level of high school (OR 1.90; 95% CI 1.31–2.77)) or < high school (OR 1.51;95% CI 0.92–2.48), were never married (OR 1.83; 95% CI, 1.17–2.86) or widowed/divorced/separated (OR 4.64; 95% CI 2.61–8.26), had one sexual partner (OR 1.16; 95% CI 0.14–9.46) or 2–5 sexual partners (OR 3.73; 95% CI 0.48–29.0) or > 5 sexual partners (OR 3.97; 95% CI 0.55–28.8) in lifetime (Table 3).

**Table 3:** Weighted Logistic Regression Models of *Chlamydia trachomatis* by Combining NHANES 2005–2010 and NHANES 2011-2016^a^.

Characteristic	OR (95% CI)	*P* value
Gender		> 0.9
Male	1 (Reference)	
Female	0.99(0.68, 1.44)	
Age, y		< 0.001
18–29	3.28(2.01, 5.35)	
30–39	1 (Reference)	
Race/ethnicity		< 0.001
Non-Hispanic White	1 (Reference)	
Non-Hispanic Black	5.01(3.14, 8.01)	
Mexican American	2.85(1.72, 4.72)	
Education		0.003
< High school	1.51(0.92, 2.48)	
High school	1.90(1.31, 2.77)	
> High school	1 (Reference)	
Marital status		< 0.001
Married/living with partner	1 (Reference)	
Never Married	1.83(1.17, 2.86)	
Widowed/divorced/separated	4.64(2.61, 8.26)	
Family poverty ratio		0.5
< 100%	1 (Reference)	
≥ 100%	1.10(0.80, 1.50)	
Age at first sex^b^, y		0.15
< 18	1.36(0.89, 2.07)	
≥ 18	1 (Reference)	
Lifetime no. sexual partners		0.005
0	1 (Reference)	
1	1.16(0.14, 9.46)	
2–5	3.73(0.48, 29.0)	
> 5	3.97(0.55, 28.8)	
Past STI diagnosis^c^		0.2
Yes	1 (Reference)	
No	1.69(0.81, 3.53)	

NHANES National Health and Nutrition Examination Survey, STI sexually transmitted infection.

^a^All estimates were weighted to be nationally representative.

^b^Defined as vaginal, oral, or anal.

^c^Participants who have been told by a doctor or other health care professional in the last 12 months that they had chlamydia or gonorrhea or have ever been told they have herpes or genital warts.

## Discussion

A nationally representative population of US were included in the present study. Our data showed that the overall prevalence of chlamydia in 2011–2016 was 1.88% among adults aged 18–39 years, which was similar to previous studies that reported a high prevalence of chlamydia^8,9^. It had a significant relative increase of 19.7% from 2005 to 2010 in which chlamydia prevalence was 1.57%. However, a previous study indicated that there was an estimated 40% reduction from 1999 to 2008^13^, our results suggested that chlamydia prevalence was 2.10% in 1999–2004, the highest among the three surveys, significantly decreased in 2005–2010, but had a re-increasing trend in recent years. Additionally, significant increases of chlamydia prevalence were observed in male and those aged 18–29 years, had > high school educational level, never married, age at first sex < 18 years, had 2–5 sexual partners in lifetime and had no past sexually transmitted diagnosis between 2005 and 2016. Multivariable logistic regression analysis demonstrated that chlamydia was more prevalent in those aged 18–29 years, being non-Hispanic Black, had high school educational level, being widowed/divorced/separated and had > 5 sexual partners.

Considerable prevalences of chlamydia infection have also been observed in other countries in recent years. Li et al. showed that chlamydia prevalence was 6.33% in female outpatients with genital tract infections based on a nationwide, multi-center and cross-sectional study between 2017 and 2018 in China^14^, and concluded chlamydia prevention strategies should incorporate both behavioral interventions and early screening programs to identify and treat individuals with genital tract infections, particularly those with risk factors. In addition, Campaner et al. performed a study of 11 years’ surveillance of chlamydia infection in Brazil and reported the overall prevalence was 2.2% in the study population^15^. Klavs et al. reported that the age-specific prevalence was the highest among individuals aged 18–24 years, 2.8% in men and 4.7% in women from a national survey in Slovenia between 2016 and 2017^16^. Therefore, chlamydia infection with high prevalence was a globally noticeable sexually transmitted disease that required primary prevention, targeted testing and effective management.

The surveillance data of CDC in the US finds higher rates of reported chlamydia among women from 2000 to 2018^1^. However, our findings contrast this in the 2011–2016 with males having a higher prevalence than females. This pronounced increase among males could be attributed to either increased transmission or improved case identification (e.g., through intensified extra-genital screening efforts) among gay, bisexual, and other men who have sex with men (MSM)^1^. Additionally, a significant advantage of population-based prevalence studies of chlamydia, such as NHANES, is the ability to accurately assess the true burden of the disease by detecting infections, whether they present with symptoms or not. Limited research has been conducted to determine the proportion of asymptomatic chlamydial infections. However, data from community screening sites in New Orleans, Louisiana, revealed that up to 77% of chlamydial infections were asymptomatic^17^. Therefore, we can monitor the chlamydia prevalence fully and accurately by combining reportable surveillance and detecting disease. Although symptoms were not specifically inquired about, making it impossible to differentiate between symptomatic and asymptomatic infections, it is crucial to measure the burden of asymptomatic infections because they are less likely to receive treatment. Untreated chlamydial infections can persist for up to one to two years, and they can be transmitted to sexual partners, increasing the risk of long-term complications in women^18^.

The NHANES results demonstrated that chlamydial infections disproportionately affect young people and non-Hispanic Black individuals. These findings were consistent with previous surveillance data and results among subgroups of the U.S. population^1,8,9^. Young individuals may have an increased risk of infection due to biological, contextual, and behavioral factors. Racial and ethnic disparities in chlamydia prevalence may be attributed to differential exposure to the disease within sexual networks, as well as limited access to routine preventative care that includes chlamydia screening and effective partner treatment. Addressing these disparities will require targeted interventions, including federally-funded programs and initiatives such as the CDC’s Community Based Approaches to Reducing Sexually Transmitted Diseases (CARS) initiative, which seeks to promote prevention and control of sexually transmitted infections through interdisciplinary interventions and enhanced community-clinical linkages.

Consistent with other studies^19,20^, we found that participants with lower educational levels were at higher risk of incident chlamydia infection. This finding may be due to that those with a higher educational level had a better sexual education and tended to avoid high-risk sexual behaviors^19,20^. Additionally, our results showed that participants who were never married or widowed/divorced/separated had a higher prevalence of chlamydia^19^. This finding may be due to that unmarried individuals are more likely to have multiple partners^21^.

The analysis presented in this report have several limitations. Firstly, the data utilized are cross-sectional, it is not possible to capture newly developed cases of chlamydia or assess the duration of chlamydia. Second, it is important to note that the prevalence estimates provided in this report may not account for chlamydial infections occurring at non-genital sites, which can also be contracted through sexual contact, such as the rectum and oropharynx. Therefore, these estimates are likely to underestimate the true burden of sexually transmitted infections. Third, it is possible that some participants may have inaccurately reported their sexual activity status, either by falsely claiming to be sexually active or by denying their sexual activity. However, the present study provided crucial observations of current epidemiology of chlamydia infection, and disparities of chlamydia in sociodemographic and lifestyle factors, which may promote future studies and public health planning.

## Conclusions

The overall chlamydia prevalence was 1.88% in 2011–2016 and 1.57% in 2005–2010, having a relative increase of 19.7% between the two surveys. Increases was especially concentrated in persons who were male, aged 18–29 years, had > high school educational level, never married, age at first sex < 18 years, had 2–5 sexual partners in lifetime and had no past sexually transmitted diagnosis. Those with high chlamydia prevalence such as sexually active young adults and Non-Hispanic Black should be screened annually so that infected persons can be diagnosed and they and their sex partners can be treated promptly.

## Data Availability

The datasets generated and/or analyzed during the current study are available in the open database NHANES website: https://wwwn.cdc.gov/nchs/nhanes/Default.aspx.
